# Mathematical model for empirically optimizing large scale production of soluble protein domains

**DOI:** 10.1186/1471-2105-11-113

**Published:** 2010-03-01

**Authors:** Eisuke Chikayama, Atsushi Kurotani, Takanori Tanaka, Takashi Yabuki, Satoshi Miyazaki, Shigeyuki Yokoyama, Yutaka Kuroda

**Affiliations:** 1 Genomic Sciences Center RIKEN, 1-7-22 Suehiro-cho, Tsurumi-ku, Yokohama 230-0045, Japan; 2 Department of Biophysics and Biochemistry, Graduate School of Science, The University of Tokyo, 7-3-1 Hongo, Bunkyo-ku, Tokyo 113-0033, Japan; 3 Department of Biotechnology and Life Science, Faculty of Technology, Tokyo University of Agriculture and Technology, 2-24-16 Naka-cho, Koganei, Tokyo 184-0012, Japan

## Abstract

**Background:**

Efficient dissection of large proteins into their structural domains is critical for high throughput proteome analysis. So far, no study has focused on mathematically modeling a protein dissection protocol in terms of a production system. Here, we report a mathematical model for empirically optimizing the cost of large-scale domain production in proteomics research.

**Results:**

The model computes the expected number of successfully producing soluble domains, using a conditional probability between domain and boundary identification. Typical values for the model's parameters were estimated using the experimental results for identifying soluble domains from the 2,032 Kazusa HUGE protein sequences. Among the 215 fragments corresponding to the 24 domains that were expressed correctly, 111, corresponding to 18 domains, were soluble. Our model indicates that, under the conditions used in our pilot experiment, the probability of correctly predicting the existence of a domain was 81% (175/215) and that of predicting its boundary was 63% (111/175). Under these conditions, the most cost/effort-effective production of soluble domains was to prepare one to seven fragments per predicted domain.

**Conclusions:**

Our mathematical modeling of protein dissection protocols indicates that the optimum number of fragments tested per domain is actually much smaller than expected *a priori*. The application range of our model is not limited to protein dissection, and it can be utilized for designing various large-scale mutational analyses or screening libraries.

## Background

Comprehensive elucidation of the functional and structural units present in the proteome is the ultimate goal in proteomics research, and it is expected to provide basic data for a rational understanding of complex biological systems. As proteomics studies are being pursued [1-5], the development of efficient methodologies for dissecting long protein sequences into their domains is becoming critical. This is because biologically important proteins are often large and are thus difficult to express, purify and characterize in a high throughput manner [6].

Experimental approaches for dissecting proteins are usually based on limited proteolysis, which has been used to explore protein domain boundaries [7]. Although experimental protein dissection methods have been extended to high throughput protocols [8-10], they remain essentially expensive and time-consuming.

Computer-aided protein dissection approaches are relatively inexpensive, and thus represent promising methodologies that have practical values in high throughput proteomics research. The strategies for predicting novel domain regions, without sequence similarity to domain databases, can be categorized into two classes. The first strategy aims at directly predicting domain regions by analyzing various sequence properties of the foldable region (e.g., see Refs [11,12]). The second strategy is to first predict the location of the domain boundaries and then use this information to infer the domain's position (e.g., see Refs [13,14]). Both strategies are essential to efficiently identify novel protein domains.

Proteomics projects require the identification of soluble and well behaved proteins enabling a fast structural/functional analysis [15]. Solubility is an important criterion strongly reflecting a protein's suitability for biophysical characterization. It can be readily monitored, and solubility assays are thus applied to large-scale studies [16]. Furthermore, when solubility is used to assess domain dissection experiments, it appears that a large fraction of soluble fragments are indeed well folded as assessed by NMR [17,18].

So far, reports of high throughput protein domain production protocols have mainly focused on their development and on optimizing individual experimental steps of the protocols. No study has mathematically modelled a protein domain production protocol in terms of a production system, and thus substantial room for cost-optimization through mathematical modeling remains available. In this report, we present a mathematical model for empirically optimizing large-scale protein domain production. Our model conceptually divides domain predictions into the prediction of the domain region and its boundary, and it computes the expected number of successfully produced soluble domains, using a conditional probability between these two events. We estimated the model parameters using the experimental results from a computer-aided identification of novel soluble protein domains from Kazusa HUGE protein sequences, in which 436 fragments, encoding 36 novel putative domains with slightly different domain boundaries, were expressed by using an E-coli-based cell-free system, and their solubilities were assessed with SDS-PAGE gels.

## Results and Discussion

### Mathematical model of protein dissection

**Figure 1:**
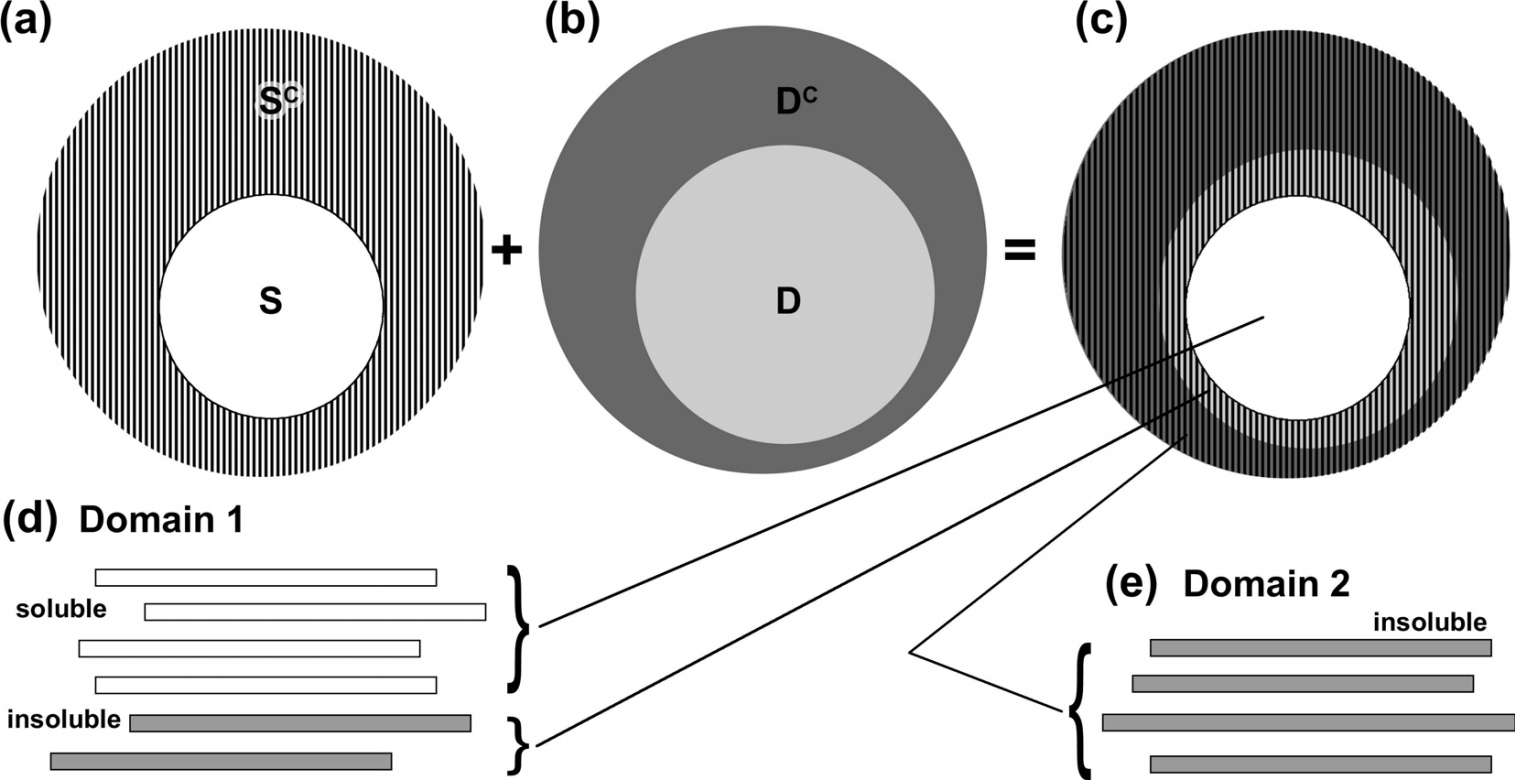
**Sets for our model**. An element of the sets in our model is an experimentally expressed protein domain fragment. (a) All elements are either soluble (S) or insoluble (S^C^) fragments. (b) Alternatively, each fragment belongs to either a soluble domain (D) or a non soluble domain (D^C^). (c) The whole set can be decomposed into four exclusive subsets: D ∩ S (white area); D ∩ S^C ^(lines on light grey area); D^C ^∩ S^C ^(lines on dark grey area); and D^C ^∩ S (not shown, since it is empty). (d) An example of the elements in the D subset. Fragments from a soluble domain (domain1) are shown. They can be classified into either the D ∩ S or D ∩ S^C ^subset (e) Fragments from a non soluble domain (domain 2) are shown. All elements belong to D^C ^∩ S.

In our mathematical model, the prediction of a protein domain is conceptually divided into two steps: A first step that predicts the existence of a domain, and a second step that predicts its boundaries (or termini). "Fragments" are domain fragments with specific termini residues, and each fragment is either soluble or insoluble. Soluble fragments and insoluble fragments belong to the soluble (S) and insoluble set (S^c^), respectively (Figure 1a). S and S^c ^are mutually exclusive sets, and S^c ^is the complement of S. We define a "soluble domain" as a domain that encodes at least one soluble fragment. A fragment that is associated with a "soluble domain" is an element of the set D (Figure 1b). According to this definition, some fragments encoding a soluble domain may be insoluble. The fragments encoding non-soluble domains, i.e., predicted domains for which all fragments are insoluble, are elements of the set D^c ^(Figure 1b and 1e). Practically, a domain is defined as non-soluble if all of the tested fragments associated with a given domain (in our experiment, 9 per domain, on average) are insoluble. D and D^c ^are also mutually exclusive sets, and their elements are fragments (not domains). The above classification yields four fragment categories (Figure 1c): S ∩ D (Soluble domain fragments with correct N and C termini), S^c ^∩ D (Insoluble fragments encoding a soluble domain, presumably because of incorrect termini), S ∩ D^c ^(Soluble fragment encoding a non-domain or a failed domain prediction; this set is obviously empty), and S^c ^∩ D^c ^(Insoluble fragment encoding a non-domain; all elements of D^c ^obviously belong to S^c^).

The probability of successfully predicting a soluble fragment, *p*(S), is expressed as(1)

since S ∩ D^c ^is empty and all sets are exclusive. *p*(D) is thus related to *p*(S) as(2)

where *p*(S|D) is the conditional probability of obtaining a soluble fragment of a soluble domain. When *p*(S) and *p*(S|D) are given, the probability of successfully predicting the existence of a soluble domain is calculated as(3)

Note that Eqs. 1-3 are direct consequences from probability rules for independent sets, without any approximations or assumptions.

In a large-scale experiment aimed at obtaining as many soluble domains as possible, cost-optimization is achieved by maximizing the number of soluble domains for a fixed number of tests. As an approximation, our model computes the expected number of producing soluble domains, *E*_domain_, by assuming average probability values over all of the protein domains examined in the experiment. Our model examines *M *protein domains and generates *N *fragments per domain. According to this model, the expected number of soluble domains, *E*_domain_, is given by:(4)

where *M *and *N *are, respectively, the number of domains and the number of fragments per domain that are assessed. When the total number of fragments (*MN*) is held *constant*, the probability of obtaining one or more soluble fragments, *P*_*N*_, is a function of *N *and *p*(S|D). The explicit form of *P*_*N *_depends on the experimental setting, and is derived in the next section for two specific cases.

We can further modify Eq. 4 to add a set-up cost related to the analysis of a new domain. The set-up cost is taken into account by expressing the total cost as *MN *+ *Mr*, where *r *is the ratio between the supplemental cost of analyzing a fragment from a new domain and that of analyzing a new fragment from the current domain. Keeping the total cost constant [*M *(*N *+ *r*) = *constant*] yields:(5)

### Derivation of *P*_*N *_for two basic experimental settings

Let us derive *P*_*N *_for two basic types of experimental settings. In the first one, the generation of *N *fragments occurs by independent events (multiple copy case). This situation occurs in genetic screening experiments, where *N *fragments per domain are randomly selected and tested, allowing multiple copies of the same fragment to be tested. In this case, the mathematical expression for *P*_*N *_is simply calculated as:(6)

where *P*_*N *_is the probability of obtaining one or more soluble fragments of a soluble domain when *N *fragments are simultaneously tested. *F *is the (average) number of all of the testable (assessable) fragments, and *f *is the (average) number of soluble fragments associated to a domain.

The second situation occurs when *N *fragments are generated, but each fragment is selected only once (single copy case). This situation occurs when the fragments are identifiable, such as in our pilot experiment with the Kazusa sequences. *P*_*N *_is derived by using a hypergeometric distribution (*P*_N_^C^), which describes the probability of obtaining no soluble elements when *N *elements are drawn without replacement from a finite population of _*F*_*C*_*N *_elements (_*m*_*C*_*n *_indicates the binomial coefficient for choosing *n *elements from *m *elements). *P*_*N *_is given by(7)

where *P*_N_^C ^is the probability of obtaining no soluble fragments when *N *fragments are tested (see Additional file 1 for detail.) A JavaScript program, implementing Eqs. 5-7, is available in Additional file 2.

### Parameter estimation from a pilot experiment with Kazusa HUGE domains

The application range of our mathematical model is not restricted to describe or analyze a specific domain prediction method (such as Armadillo[19], or PRODOM[20]), or experimental procedure (E-coli strains, or cell free systems). The specific settings/protocols are taken into account by adjusting or optimizing the values of the model's parameters. Here, we will estimate typical values for the parameters using the solubility of protein domains predicted in the Kazusa HUGE protein sequences [21].

**Figure 2:**
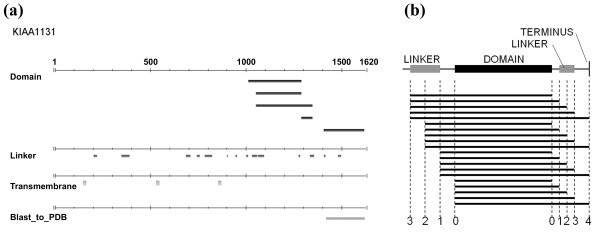
**Design of domain fragments**. (a) Snap shot of the ProteoMix for the Kazusa HUGE KIAA1131 sequence. The results of PASS (domain predictions, Domain), DLiP and DLP (domain linker predictions, Linker), HMMTOP and MEMSAT (transmembrane predictions, Transmembrane), and BLAST to PDB sequences (homology to structurally known sequences, Blast_to_PDB) are shown. (b) Domain fragment design scheme. For each dissected domain, the corresponding linker or the N- or C-terminus of the full-length sequence that is in the N- or C-terminal end of the domain is used to determine the termini of the fragments. Three cleavage sites are defined along the linker region. The first one is at the end of the predicted linker on the PASS-predicted domain side (cleavage site 1), the second one is located in the middle of the linker region (cleavage site 2), and the third one is at the end of the linker region, opposite from the domain side (cleavage site 3). In addition, we designed a cleavage site at the terminal ends of the PASS-predicted domain (cleavage site 0) and at either the N or C terminus of the protein (cleavage site 4, in case it was close to the C- or N- terminus of the domain). Some cleavage sites were not defined: for example, when the length of a linker was too short to determine its center, the central cleavage site was omitted. The number of generated fragments was regarded as sufficient for assessing the presence of a cleavable site and for estimating the model's parameters.

**Figure 3:**
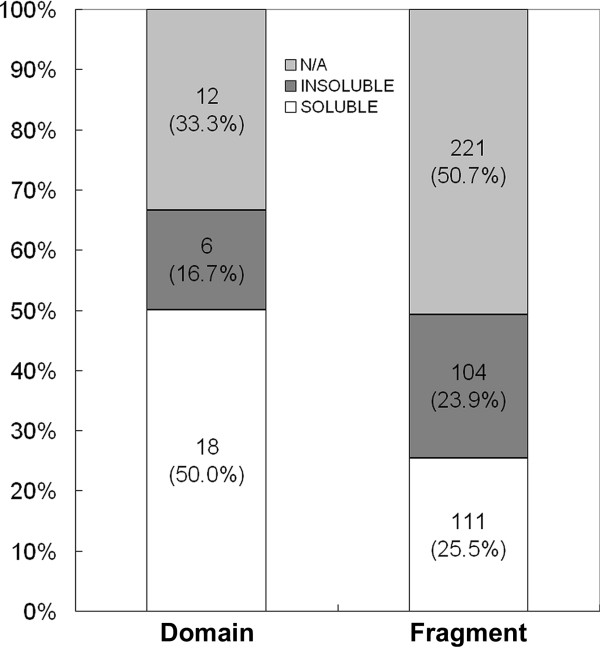
**Summary of the solubility experiment**. Soluble (SOLUBLE), insoluble (INSOLUBLE), and not applicable (N/A) domains (left bar) and the corresponding fragments (right bar) are shown. N/A includes all kinds of experimental incompleteness, such as insufficient PCR, wrong cDNA sequences, no expression or ambiguous SDS-PAGE results.

We first identified 36 putative domains using ProteoMix [22] (Figure 2a), and for each domain, we expressed several fragments with different N and/or C-terminal residues distributed over the predicted termini window (Figure 2b). This yielded a total of 436 fragments; namely, 12 fragments per domain were generated, on average, to probe the domain termini. Among the 436 fragments, 215 (corresponding to 24 domains) were expressed correctly and were eventually assessed (Additional file 3); 111 fragments (encoding 18 domains) were soluble, and 104 fragments (encoding 6 domains) were insoluble (Figure 3).

**Figure 4:**
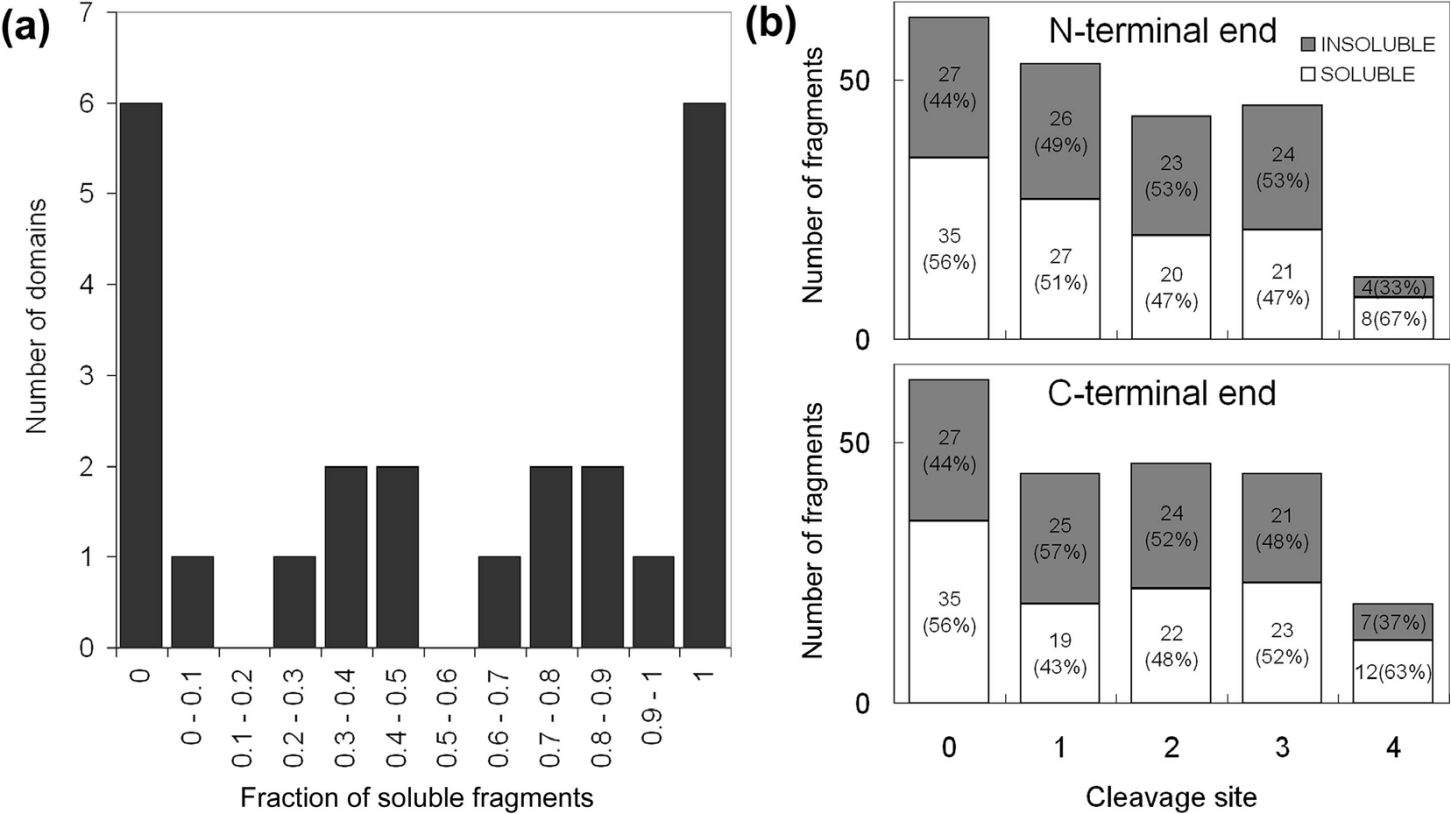
**Soluble fragments for individual domains and cleavage sites**. (a) Fractions of soluble fragments for individual domains. The horizontal axis represents the ratio of soluble fragments to assessed fragments for each of the 24 analyzable domains. (b) Distribution of the cleavage sites yielding soluble/insoluble fragments. The numbers of soluble and insoluble fragments are shown by white and dark gray bars, respectively. The N- (upper panel) and C-terminal (bottom panel) cleavage sites of the fragments are classified into sites 0-4, as defined in Figure 2b.

For the purpose of discussion, let us estimate the parameters using the 215 fragments corresponding to the 24 domains that expressed correctly. Among these domains, 75% (18/24), and 52% (111/215; which corresponds to *p*(S) and *p*(S^C^) = 0.48; Table 1) of the fragments were soluble when 9 fragments were tested on average (instead of 12 when all 436 of the fragments are considered). *p*(S|D), which is the conditional probability of correctly predicting a soluble fragment of a soluble domain, is 0.63 (111 fragments/175 fragments). The calculation of *p*(D) from Eq. 3, using the obtained values of *p*(S) and *p*(S|D), generates 0.81. Substituting 0.81 and 9 for *p*(D) and *N*, respectively, in Eq. 4, yields *E*_domain_/*M *as 0.81, which is equal to *p*(D), within rounding error, since *P*_*N *_is almost 1 when *N *= 9. The discrepancy from the above experimental *E*_domain_/*M *value of 0.75 (Figure 3), is due to the use of average values. Indeed, for 6 domains, all of the fragments were soluble, and 12 domains yielded a mixture of soluble and insoluble fragments (Figure 4a). However, the corrections appear to be modest (less than 10%) and will not significantly affect the following discussion (the calculation with non-averaged *f *and *F *values is discussed in Additional file 1).

**Table 1:** Estimated model parameters

	D	D^C^	ALL
S	0.52 ± 0.03	0.00 ± 0.00	0.52 ± 0.03^a^

S^C^	0.29 ± 0.03	0.19 ± 0.03	0.48 ± 0.03^b^

ALL	0.81 ± 0.03^c^	0.19 ± 0.03^d^	1.00

a: *p*(S); b: *p*(S^C^); c: *p*(D); d: *p*(D^C^); S, S^C^, D, D^C^: see the main text; ALL: All the considered set; *p*(S ∩ D) (upper left cell): The probability of predicting a soluble fragment of a soluble domain, similarly for the S^C ^∩ D (middle left), S ∩ D^C ^(upper center), and S^C ^∩ D^C ^(middle center); Standard deviations were computed by bootstrapping, in which 1,000 sets of 215 randomly selected fragments were sampled.

Let us perform a simple mathematical simulation to assess the discrepancy between the above experimental and calculated values of 0.75 and 0.81. The discrepancy can be resolved by setting the number of tested fragments per domain to, for example, 10, and by uniformly using for all domains the above experimentally determined average values of 0.75 and 0.52 for *p*(D) and *p*(S), respectively. As a result, 240 fragments are produced, corresponding to 24 domains, of which 6 do not yield any soluble fragment, since *p*(D) = 0.75. Since *p*(S) = 0.52, 124.8 fragments are soluble. These soluble fragments are uniformly distributed among the 18 domains, and thus 6.93 (7) fragments per domain out of 10 fragments are soluble, which yields 0.693 for *p*(S|D). Using Eq. 3, *p*(D) = 0.52/0.693 = 0.75, and thus *p*(D) *P*_*N *_= 0.75 * {1 - (1 - 0.693)^10 ^= 0.75, which is equal, within rounding error, to the experimentally determined value of 0.75 (18 domains out of 24).

In the present experiment, 12 domains (221 fragments) could not be analyzed because of failure to express a fragment with a correct molecular weight, or to assess the solubility, which obviously indicates that the efficiency of our automated protein expression system has room for improvement. These 12 domains can be included in the analysis and will give lower (or upper) limits by considering them as insoluble (or soluble) domains. The inclusion yields 50% (18/36) and 25% (111/436) for, respectively, the lower probability limit of predicting the domain existence and that of predicting a soluble fragment for 12 tested fragments, on average. Similarly, some or all of the 6 domains that were considered insoluble in the above discussion might turn out to be soluble if more fragments were tested.

### Effect of the cleavage site on the solubility of the dissected domain

The effect of the cleavage site on the solubility of the dissected domain was also examined (Additional file 3). All of the fragments corresponding to 6 domains (KIAA0190.21-227, KIAA0309.483-717, KIAA1142.35-97, KIAA1256.13-117, KIAA1416.55-202, and KIAA1459.745-848) were soluble (all-soluble class); while KIAA0067.1143-1295, KIAA0175.480-651, KIAA0190.436-653, KIAA0277.120-349, KIAA0641.76-352, and KIAA1338.180-406 were insoluble (all-insoluble class), indicating a failed domain prediction. On the other hand, the solubility of the remaining 12 domains was dependent on the position of the cleavage site, and both soluble and insoluble fragments were produced, depending on slight shifts of the cleavage residue at each of the N- and C- domain terminal ends (soluble+insoluble class). The effects of the cleavage site within the window were also examined. Except for site 4, which corresponded to either the N or C protein terminus, no differences in the yields of soluble fragments were observed (Figure 4b). Note that the small number of cleavage at site 4 simply results from the fact that not all domains were located at the N or C protein terminus.

### Optimum fragment number per domain

**Figure 5:**
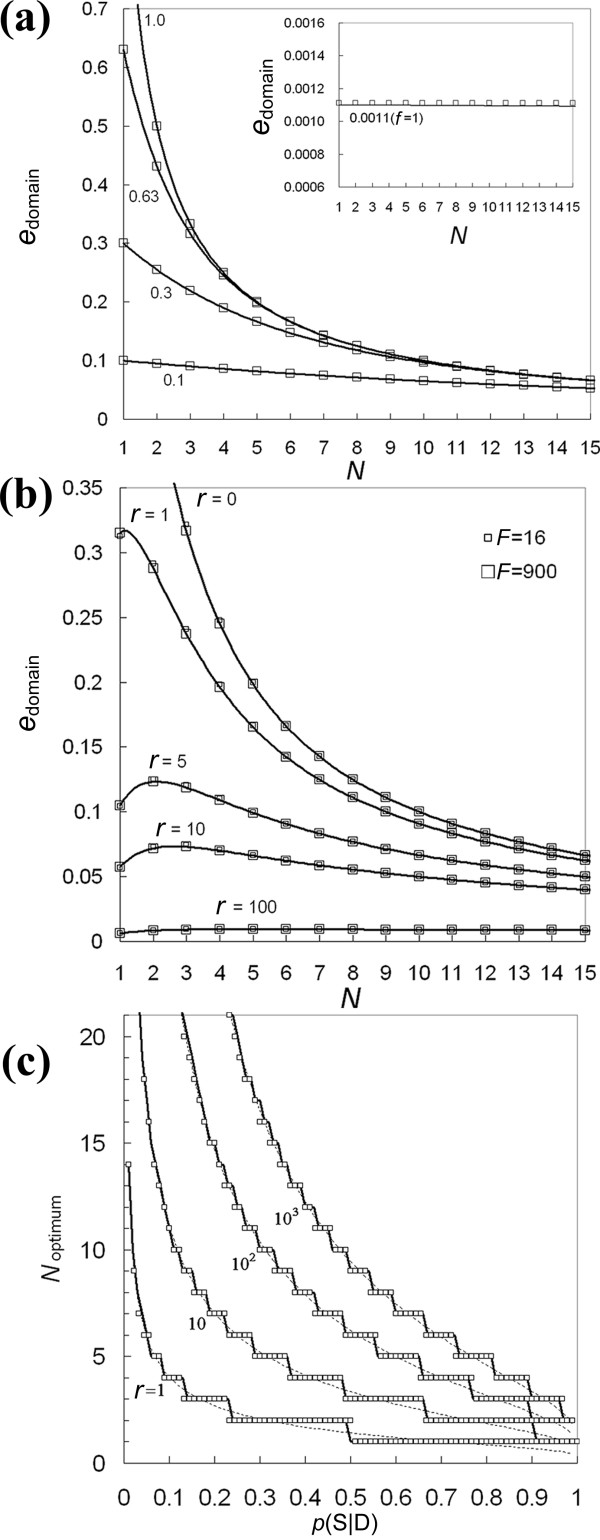
**Dependence of *e*_domain _on *N***. *e*_domain _and *N*_optimum _were calculated for the multiple and single copy cases with, respectively, Eq. 6 (solid lines) and Eq. 7 (symbols). (a) *e*_domain _is represented as a function of *N *for *p*(S| D) = 1, 0.63, 0.3, and 0.1. The curve for the special case of *f *= 1 [*p*(S| D) = 0.0011] is shown in the inserted panel, with vertical scale magnified 300 times. (b) Curves for *r *> 0 and *F *= 16 and 900. *e*_domain _are shown for *r *= 0, 1, 5, 10, and 100 with *p*(S| D) = 0.63. *N*_optimum _> 1 is clearly apparent. (c) *N*_optimum _is shown as a function of *p*(S| D) for *r *= 1, 10, 100, and 1,000. *N*_optimum _can be any positive number. *N*_optimum _for the single copy case are discrete in *N *(symbols), while those for the multiple copy case are continuous (dotted lines). Discretization of the single copy model (solid lines) caused a small discrepancy at the very edge of switching a step of solutions.

Let us use our model to analyze protein dissection experiments with different settings. Since *p*(D) is independent of *N*, we can simplify Eq. 5, and examine the normalized expected number of soluble domains, *e*_domain_, instead of *E*_domain_:(8)

where *P*_*N *_can be computed with either Eq. 6 or 7, since no noticeable difference will occur for practical purposes (for most cases, within a few percent error; see Figure 5). When no set-up cost is present (*r *= 0), *e*_domain _is a monotone decreasing function of *N *(Figure 5a). Thus, one fragment per predicted domain will optimize the number of soluble domains (*N*_optimum _= 1). This is analytically demonstrated by showing that *N*_optimum _is the solution of a transcendental equation with a unique solution (A mere exception occurs for *f *= 1 when the single copy per fragment model is used; Figure 5a and Additional file 1).

Set-up costs are typically generated by the purchase of new chemicals associated with the examination of a new domain, and may, for example include the clone's cost, from which the domain fragments are prepared by PCR. Large *r *values may occur in a genetic screening-type experiment, where the cost of assessing a new fragment is small as compared to that of starting a new experiment with a new domain. For *r *> 0, *e*_domain _is not a monotone decreasing function of *N*, but reaches a maximum at *N *= *N*_optimum _(Figure 5b). The value of *N*_optimum _increases for increasing values of *r *(Figure 5b).

As an example, using the values derived from our pilot experiment [*p*(S|D) = 0.63 (= *f*/*F*)] and assuming that *r *= 10, we find that testing three fragments per predicted domain would yield more soluble domains than just one, for the same total cost (Figure 5b). Note that, as *r *increases, the peak broadens and the maximum *e*_domain _value, *e*_domain _(*N*_optimum_), becomes smaller (Figure 5b). Thus, in practical terms, the *e*_domain _dependence on *N *is small for large *r *and it becomes less important to accurately determine *N*_optimum_. Finally, we note that in Eq. 6 (multiple copy model), *e*_domain _depends on the ratio of *f *and *F*, which is the probability of finding the correct cleavage sites, but it does not depend directly on *F *alone, which is the total number of possible cleavage sites. The direct dependency on *F *alone is also minimal when the single copy model (Eq. 7) is used (Figure 5b).

Insight into a wide range of experimental settings can be obtained by analyzing the behavior of *N*_optimum _for several values of *p*(S|D) and *r *(Figure 5c). For example, we find that *N*_optimum _increases with decreasing values of *p*(S|D), which is intuitively sensible, since a smaller *p*(S|D) requires a larger number of trials for finding the termini residues that yield a soluble domain fragment. *N*_optimum _is between 1 and 4 for *r *= 1 and a broad range of *p*(S|D) > 0.1, which covers most experimental settings including our pilot experiment using Kazusa sequences. A *p*(S|D) > 0.1 would also cover typical domain prediction tools such as Armadillo prediction, which has a *p*(S|D) value estimated by cross validation method between 0.3 and 0.5 [19]. Finally, for a typical value of *r *= 10 and *p*(S|D) > 0.5, *N*_optimum _is between 1 and 3; and for *p*(S|D) = 0.63, *N*_optimum _is 7 fragments, even for *r *= 1,000 (i.e., a very large set-up cost).

## Conclusions

We have presented a novel mathematical formulation for optimizing large-scale protein domain production experiments. Our model demonstrated that the testing of one to seven fragments per domain will fit most high throughput protein domain production experiments. The probabilistic approach presented here is not limited to protein domain production, and it can be readily modified and applied for designing various types of large-scale mutational analyses or screening libraries.

## Methods

### Computational protein dissection

Domains were computationally predicted from 2,032 Kazusa HUGE [21] protein sequences (KIAA0001-KIAA2033), using ProteoMix [22], an integrated protein sequence analysis system. The ProteoMix analysis included PASS [23] for domain predictions, and DLiP [24] and DLP [13,25] for domain linker predictions. PASS analyses were performed with default parameters (E-value = 1e-7, Cut-off homologues = 10); DLP with default (Threshold = 0.5, Cut-off = 0.5, Ignored terminal length = 0, Window = 19, Minimum difference = 0.05); and DLiP with no BLAST option. The other tools were HMMTOP [26] and MEMSAT [27] for trans-membrane region prediction, and BLAST [28] for removing domains homologous to structurally known protein sequences derived from the Protein Data Bank [29]. We obtained 269 putative domains according to the following rules: A putative domain was identified when it was a PASS predicted domain region whose boundary overlapped either with one of the full-length protein termini, or with a domain boundary predicted with either DLiP and DLP results (predicted domain linker regions) within ± 25 residues. We also required that the putative domain did not include transmembrane regions, as predicted by HMMTOP or MEMSAT with default parameters, to remove the inherently insoluble domains; and putative domains with sequence identities higher than 30% to PDB protein sequences were also removed. Finally, we choose 27 domains by visually inspecting the consistency among the prediction's tools, domain's, and domain linker's sizes. In addition, we completed this set with 9 domains predicted in one of the putative multi-domain protein sequences that contained one of the above 27 domains. This yielded 36 domains that were assessed experimentally. For each of the 36 dissected domains, a maximum of 20 fragments per domain, resulting in a total of 436 fragments, were designed by combining the results of PASS, DLiP, and DLP, and our termini selection rule (see Figure 2b). This resulted in a 30 residue N- or C- terminal end window (corresponding to 900 fragments per domain) on average, from which the termini residues were selected.

### Experimental assessment

The cDNA clones for the selected 36 protein domains (436 fragments) were kindly provided by Kazusa DNA Research Institute (Kisaradsu, Japan). The corresponding protein fragments were expressed using an E-coli based cell-free system and were purified as described [30]. The fragments were classified as soluble, insoluble, and not applicable, according to an SDS-PAGE analysis of the supernatant and precipitate fractions. The soluble fragments were defined as the fragments that remained in the supernatant after centrifugation. The fragments that were present in the SDS-PAGE of the precipitate, but absent from the supernatant after centrifugation, were defined as insoluble fragments. All other fragments were classified as not applicable, which included all kinds of experimental obstacles, such as unsuccessful PCR, wrong cDNA sequences, no expression or ambiguous SDS-PAGE results.

## Authors' contributions

EC, AK, TY, SY, and YK designed the research. YK and EC derived the mathematical forms. TT, SM, AK, and EC developed the analysis programs. TY performed the experiments. EC and AK analyzed the data. EC and YK wrote the paper.

## Supplementary Material

Additional file 1 **Additional methods**. An extension for handling the distribution of *p*(S|D) with the multiple copy model; Basic properties of *P*_*N*_; Intuitive Derivation of *P*_*N *_for the single copy per fragment case; Direct Derivation of *P*_*N *_for the single copy per fragment case using the hypergeometric distribution. Click here for file

Additional file 2 **Calculator of the expected number of soluble domains**. A JavaScript program, implementing Eqs. 5-7. Click here for file

Additional file 3 **Figure S1: List of expressed domains**. List of expressed domains in our pilot example. Computationally dissected protein domains and their experimentally assessed solubilities. Click here for file
