# Supplementary material for: Mathematical model for empirically optimizing large scale production of soluble protein domains
Source: BMC Bioinformatics. 2010 Mar 1;11:113. doi: 10.1186/1471-2105-11-113 (PMC2843616; doi:10.1186/1471-2105-11-113)
Supplement: Additional file 1 — Additional methods. An extension for handling the distribution of p(S|D) with the multiple copy model; Basic properties of PN; Intuitive Derivation of PN for the single copy per fragment case; Direct Derivation of PN for the single copy per fragment case using the hypergeometric distribution. [file 1471-2105-11-113-S1.PDF]

Supplementary Methods

An extension for handling the distribution of p(S|D) with the multiple copy model

p(S|D) might vary from one domain to another. It is indeed possible to use a different p(S|D) value for each domain, instead of an average value as in Eq. 7, $\overline{p(\mathrm{S} \mid \mathrm{D})} = \sum_{i=1}^{M} p_i(\mathrm{S} \mid \mathrm{D}) / M$. p(S|D) for domain i is denoted by $p_i(\mathrm{S} \mid \mathrm{D})$. Assuming p(D) is constant, the difference between

$$E_{\mathrm{domain}}(p_i(\mathrm{S} \mid \mathrm{D})) = p(\mathrm{D}) \sum_{i=1}^{M} \left[1 - \{1 - p_i(\mathrm{S} \mid \mathrm{D})\}^N \right] \quad \text{(A1)}$$

and

$$E_{\mathrm{domain}}\left(\overline{p(\mathrm{S} \mid \mathrm{D})}\right) = p(\mathrm{D}) M \left[1 - \left\{1 - \overline{p(\mathrm{S} \mid \mathrm{D})}\right\}^N \right] \quad \text{(A2)}$$

is derived as follows. Using

$$\begin{aligned} g(p, N) &= 1 - (1-p)^N \\ &= 1 - \left\{1 - Np + {}_N C_2 p^2 - {}_N C_3 p^3 + \cdots + (-1)^{N-1} {}_N C_{N-1} p^{N-1} + (-1)^N p^N \right\} \\ &= Np - {}_N C_2 p^2 + {}_N C_3 p^3 + \cdots - (-1)^{N-1} {}_N C_{N-1} p^{N-1} - (-1)^N p^N \end{aligned} \quad \text{(A3)}$$

with p as p(S|D) and dividing (A1 – A2) by *constant* p(D), we obtain

$$\begin{aligned} &\frac{E_{\mathrm{domain}}(p_i(\mathrm{S} \mid \mathrm{D})) - E_{\mathrm{domain}}\left(\overline{p(\mathrm{S} \mid \mathrm{D})}\right)}{constant\ p(\mathrm{D})} \\ &= e_{\mathrm{domain}}(p_i(\mathrm{S} \mid \mathrm{D})) - e_{\mathrm{domain}}\left(\overline{p(\mathrm{S} \mid \mathrm{D})}\right) \\ &= \frac{1}{N+r}\left[\frac{1}{M} \sum_{i=1}^{M} \left[1 - \{1 - p_i(\mathrm{S} \mid \mathrm{D})\}^N \right] - \left[1 - \left\{1 - \overline{p(\mathrm{S} \mid \mathrm{D})}\right\}^N \right] \right] \\ &= \frac{1}{N+r}\left\{ \overline{g(p, N)} - g(\overline{p}, N) \right\}. \end{aligned} \quad \text{(A4)}$$

The equation (A4), which yields the difference of the expected number of obtaining different domains between extended handling of the distribution and our approximated model, becomes zero if N goes to infinity. The difference is also 0 if $N = 1$. Furthermore, simulations using our experimentally observed distribution $\{p_i\}$ in Fig. 4a**,** excluding the fragments in the subset D^C (Fig. 1c), resulted in $\overline{p(\mathrm{S} \mid \mathrm{D})}$ = 0.71. Substituting this into A4, revealed that $e_{\mathrm{domain}}(p_i(\mathrm{S} \mid \mathrm{D}))$ was smaller than $e_{\mathrm{domain}}(\overline{p(\mathrm{S} \mid \mathrm{D})})$ by 0.044, 0.030, 0.0033, and 0.0030 for (N, r) = (2, 0), (2, 1), (10, 0), and (10, 1), respectively, which correspond to errors of 3 to 10% for the value of $e_{\mathrm{domain}}(\overline{p(\mathrm{S} \mid \mathrm{D})})$.

Basic properties of P_N

In practical terms, the values computed using Eqs. 6 and 7 are overlapping (for most values, within a few percent error; see also Fig. 5). The virtue of Eq. 6 is its simplicity, and in its continuous form it can be readily differentiated. N_{optimum}, which is the value of N that maximizes E_{domain}, is thus obtained by differentiating Eq. 6 with respect to N and equating it to 0, which yields

$$\exp(aN) = aN + ar + 1, \tag{A5}$$

where $a = -\log\{1 - p(\mathrm{S} \mid \mathrm{D})\} = -\log\{1 - f / F\} \geq 0$. The left-hand side is an exponentially increasing function of N, and the right-hand side is a linearly increasing function of N with an ordinate of $1+ar$. This implies that Eq. A5 has only a single solution: if the experimental set-up cost (r) is nil, then the solution to Eq. A5 is N=0 because ar = 0. Thus, if no set-up costs are incurred upon the examination of a new domain, then the most effective way is to test one fragment per domain (since zero is not a realistic solution). This is clearly illustrated in Fig. 5a, which shows that P_N is a monotone decreasing function of N.

One peculiar situation arises in the single copy model for f=1 and r=0 (Eq. 7, with no set-up cost). Eq. 7 becomes

$$E_{\mathrm{domain}}(f = 1, r = 0) = \frac{\mathrm{constant}}{N} p(\mathrm{D})\left\{1 - \frac{(F-1)!(F-N)!}{(F-1-N)!F!}\right\}$$
$$= \frac{\mathrm{constant}}{N} p(\mathrm{D})\left\{1 - \frac{F-N}{F}\right\} = \frac{\mathrm{constant}}{N} p(\mathrm{D})\frac{N}{F}$$

$$= \frac{\text{constant}}{F} p(\mathrm{D})$$

in which, surprisingly, the dependence on N vanishes. Although this is a very specific case, it is noteworthy that this situation is not reproduced with Eq. 6, where multiple copies of the same fragments can be generated. This difference arises because in the single copy model, the chances of producing a soluble fragment improve with increasing N, since each fragment is tested only once. In practical terms, this means that for small f (small number of soluble fragments) it is worth increasing the number of fragments per domain (N). On the other hand, when a large number of soluble fragments are available, only a few fragments should be tested before moving to a new domain.

Intuitive Derivation of P_N for the single copy per fragment case.

The calculation of P_N is equivalent to the classical problem of computing the probability of drawing a black marble from a finite set of black and white marbles. It is understandable by considering a probability tree, representing the random generation of a soluble (S) or insoluble (I) fragment. The outcome of N trials is denoted by a string of Is and Ss, such as SSI. For example, the possible outcomes for N = 2 are SS, SI, IS, and II. The expressions for A, the probability of obtaining one or more soluble fragments per domain, are described as follows. For N = 1,

$$P_N = P_N(\mathrm{S}) = \frac{f}{F}.$$

For N = 2,

$$P_N = P_N\,(\mathrm{SS}) + P_N\,(\mathrm{SI})$$

$$+ P_N\,(\mathrm{IS})$$

$$= \frac{f}{F} + \frac{F-f}{F} \cdot \frac{f}{F-1},$$

and for N = 3,

$$P_N = P_N\,(\mathrm{SSS}) + P_N\,(\mathrm{SSI}) + P_N\,(\mathrm{SIS}) + P_N\,(\mathrm{SII})$$

$$+ P_N\,(\mathrm{ISS}) + P_N\,(\mathrm{ISI})$$

$$+ P_N\,(\mathrm{IIS})$$

$$= \frac{f}{F} + \frac{F-f}{F} \cdot \frac{f}{F-1} + \frac{F-f}{F} \cdot \frac{F-f-1}{F-1} \cdot \frac{f}{F-2},$$

where the terms are classified by the position of the first appearance of S. For example, the first term f / F is derived from P_N (SSS), P_N (SSI), P_N (SIS), and P_N (SII), in which S appears in the first position; the second term is derived from P_N (ISS) and P_N (ISI), in which S is at the second position; the third term is derived from P_N (IIS), in which S is at the third position. N determines the size of a denominator, and it is expressed as $F(F-1)(F-2)\cdots(F-N+1)$. Finally, we obtain:

$$P_N = \frac{f}{F} + \frac{F-f}{F} \cdot \frac{f}{F-1} + \cdots + \frac{F-f}{F} \times \cdots \times \frac{F-f-N+2}{F-N+2} \frac{f}{F-N+1}$$

$$= \sum_{i=0}^{N-1} \frac{(F-f)!(F-i)!\,f}{L!(F-f-i)!(F-i)}, \qquad \text{(A6)}$$

where i represents the (i + 1)-th generation of a fragment. The condition $F-f-i \geq 0$ is required for each term, which is limited by the number of insoluble fragments, $F - f$, because the terms, except for the first term, suppose the existence of more than one insoluble fragment. If $F-f-i \geq 0$ does not hold for a term, then it is 0.

Direct derivation of P_N for the single copy per fragment case using the hypergeometric distribution

The hypergeometric distribution is defined as

$$\frac{\left({}_{f}C_{s}\right) \times \left({}_{F-f}C_{N-s}\right)}{{}_{F}C_{N}}, \qquad \text{(A7)}$$

where s is the number of successes in a sequence of N draws, F is the total number, and f is the number of successes in the total. It represents a probability of the occurrence of s successes in a sequence of N draws without replacement. In our situation for the single copy per fragment case, we need P_N^C (see Eq. 7 in the main text) and thus s is substituted by 0 in A7. It becomes,

$$P_N = 1 - P_N^C = 1 - \frac{{}_{F-f}C_N}{{}_F C_N} = 1 - \frac{(F-f)!(F-N)!}{(F-f-N)!F!} \qquad \text{(A8)}$$

with, $P_N^C = \frac{{}_f C_s \times {}_{F-f} C_{N-s}}{{}_F C_N} = \frac{{}_f C_0 \times {}_{F-f} C_N}{{}_F C_N} = \frac{{}_{F-f} C_N}{{}_F C_N}$.

Eq. A8 is equivalent to A6 as demonstrated below: P_N is decomposed as the sum of *i*-th series. An *i*-th term represents the probability of the occurrence that first $(i-1)$ draws are all failures, an *i*-th draw is a success, and the rest of $(N-i)$ draws are unspecified.

Equation A8 is proved as follows:

$$\sum_{i=0}^{N-1} \frac{(F-f)!(F-i)!f}{F!(F-f-i)!(F-i)} = \frac{(F-f)!f}{F!} \sum_{i=0}^{N-1} \frac{(F-i)!}{(F-f-i)!(F-i)}$$

$$= \frac{(F-f)!f(f-1)!}{F!(f-1)!} \sum_{i=0}^{N-1} \frac{(F-i)(F-i-1)!}{(F-i-1-(f-1))!(F-i)} = \frac{(F-f)!f!}{F!(f-1)!} \sum_{i=0}^{N-1} \frac{(F-i-1)!(f-1)!}{(F-i-1-(f-1))!(f-1)!}$$

$$= \frac{(F-f)!f!}{F!} \sum_{i=0}^{N-1} \frac{(F-i-1)!}{(F-i-1-(f-1))!(f-1)!} = \frac{1}{{}_F C_f} \sum_{i=0}^{N-1} {}_{F-i-1}C_{f-1}$$

$$= \frac{1}{{}_F C_f} \left({}_{F-1}C_{f-1} + {}_{F-2}C_{f-1} + {}_{F-3}C_{f-1} + \cdots + {}_{F-N}C_{f-1} \right)$$

Using A9 described below, we can obtain

$$\frac{1}{{}_F C_f} \left({}_{F-1}C_{f-1} + {}_{F-2}C_{f-1} + {}_{F-3}C_{f-1} + \cdots + {}_{F-N}C_{f-1} \right) = \frac{1}{{}_F C_f} \left({}_F C_f - {}_{F-N} C_f \right)$$

and therefore

$$\sum_{i=0}^{N-1} \frac{(F-f)!(F-i)!f}{F!(F-f-i)!(F-i)} = \frac{1}{{}_F C_f} \left({}_F C_f - {}_{F-N} C_f \right) = 1 - \frac{{}_{F-N} C_f}{{}_F C_f} = 1 - \frac{(F-N)!}{(F-N-f)!f!} \frac{(F-f)!f!}{F!}$$

$$= 1 - \frac{(F-N)!}{(F-N-f)!} \frac{(F-f)!}{F!} = 1 - \frac{(F-f)!}{(F-f-N)!N!} \frac{N!(F-N)!}{F!} = 1 - \frac{{}_{F-f} C_N}{{}_F C_N}.$$

(Q.E.D.)

$${}_F C_f - {}_{F-N} C_f = {}_{F-1}C_{f-1} + {}_{F-2}C_{f-1} + {}_{F-3}C_{f-1} + \cdots + {}_{F-N}C_{f-1} \qquad \text{(A9)}$$

is obtained by repeatedly applying a standard formula,

$${}_F C_f = {}_{F-1} C_f + {}_{F-1} C_{f-1},$$

to ${}_{F}C_{f}$ as follows:

$$
\begin{aligned}
{}_{F}C_{f} &= {}_{F-1}C_{f} + {}_{F-1}C_{f-1} \\
&= {}_{F-2}C_{f} + {}_{F-2}C_{f-1} + {}_{F-1}C_{f-1} \\
&= {}_{F-3}C_{f} + {}_{F-3}C_{f-1} + {}_{F-2}C_{f-1} + {}_{F-1}C_{f-1} \\
&= {}_{F-4}C_{f} + {}_{F-4}C_{f-1} + {}_{F-3}C_{f-1} + {}_{F-2}C_{f-1} + {}_{F-1}C_{f-1} \\
&= {}_{F-N}C_{f} + {}_{F-N}C_{f-1} + {}_{F-N-1}C_{f-1} + \cdots + {}_{F-3}C_{f-1} + {}_{F-2}C_{f-1} + {}_{F-1}C_{f-1}.
\end{aligned}
$$

There are (N + 1) terms in the right hand side of the last expression and transposing the first term to the left hand side is A9.
