# Supplementary material for: Mathematical model for empirically optimizing large scale production of soluble protein domains
Source: BMC Bioinformatics. 2010 Mar 1;11:113. doi: 10.1186/1471-2105-11-113 (PMC2843616; doi:10.1186/1471-2105-11-113)
Supplement: Additional file 2 — Calculator of the expected number of soluble domains. A JavaScript program, implementing Eqs. 5-7. [file 1471-2105-11-113-S2.HTML]

Mathematical Model for Empirically Optimizing Large Scale Production of Soluble Protein Domains


**Mathematical Model for Empirically Optimizing Large Scale Production of Soluble Protein Domains**   
Eisuke Chikayama, Atsushi Kurotani,
Takanori Tanaka, Takashi Yabuki, Satoshi Miyazaki, Shigeyuki Yokoyama, and Yutaka Kuroda, *BMC Bioinformatics*

---

  
Enter parameters and push **Calculate** button.

0 <= *p*(D) <= 1

*f* >= 0

*F* >= 0

*r* >= 0

*constant* >= 0

*N* >= 0 from
to

You can copy and paste
to MS-Excel:
