# Supplementary material for: Mathematical model for empirically optimizing large scale production of soluble protein domains
Source: BMC Bioinformatics. 2010 Mar 1;11:113. doi: 10.1186/1471-2105-11-113 (PMC2843616; doi:10.1186/1471-2105-11-113)
Supplement: Additional file 3 — Figure S1: List of expressed domains. List of expressed domains in our pilot example. Computationally dissected protein domains and their experimentally assessed solubilities. [file 1471-2105-11-113-S3.PDF]

NO.	KIAA code	DOMAIN	FRAGMENT	LENGTH	SOLUBLE
1	KIAA0067	1143-1295	1143-1295	153	-
2			1143-1300	158	-
3	KIAA0175	480-651	480-651	172	-
4			482-651	170	-
5			486-651	166	-
6	KIAA0190	21-227	1-201	201	+
7			1-227	227	+
8		335-436	324-430	107	+
9			324-436	113	+
10			324-440	117	+
11			324-449	126	+
12			335-430	96	+
13			335-436	102	+
14			335-440	106	+
15			341-430	90	+
16			341-436	96	+
17			341-440	100	+
18			341-449	109	-
19			357-430	74	+
20			357-436	80	+
21			357-440	84	+
22			357-449	93	-
23		436-653	431-653	223	-
24		646-808	635-808	174	-
25			635-813	179	+
26			643-808	166	-
27			643-813	171	+
28			646-808	163	-
29			646-813	168	-
30			651-808	158	-
31			651-813	163	-
32	KIAA0207	72-151	1-151	151	+
33			1-158	158	+
34			1-166	166	-
35			1-173	173	+
36	KIAA0242	133-310	119-310	192	+
37			119-315	197	+
38			124-310	187	+
39			124-315	192	+
40			129-310	182	-
41			129-315	187	-
42			133-310	178	-
43			133-315	183	-
44	KIAA0277	120-349	61-328	268	-
45			61-331	271	-
46			61-334	274	-
47			61-349	289	-
48			65-328	264	-
49			65-331	267	-
50			65-334	270	-
51			65-349	285	-
52			68-328	261	-
53			68-331	264	-
54			68-334	267	-
55			68-349	282	-
56			120-328	209	-
57			120-331	212	-
58			120-334	215	-
59			120-349	230	-
60	KIAA0309	483-650	446-647	202	-
61			446-650	205	-
62			446-658	213	-
63			446-668	223	-
64			483-647	165	+
65			483-650	168	+
66			483-658	176	+
67			483-668	186	+
68		483-717	432-708	277	+
69			432-717	286	+
70			432-719	288	+
71			432-729	298	+
72			446-708	263	+
73			446-717	272	+
74			446-719	274	+
75			446-729	284	+
76			483-708	226	+
77			483-717	235	+
78			483-719	237	+
79			483-729	247	+
80	KIAA0358	927-1118	923-1118	196	+
81			923-1139	217	-
82			923-1142	220	-
83			923-1144	222	-
84			927-1118	192	+
85			927-1139	213	-
86			927-1142	216	-
87			927-1144	218	+
88			934-1118	185	+
89			934-1139	206	-
90			934-1142	209	-
91			934-1144	211	-
92			944-1118	175	+
93			944-1139	196	-
94			944-1142	199	-
95			944-1144	201	-
96	KIAA0537	438-693	418-693	276	+
97			418-698	281	+
98			427-693	267	+
99			427-698	272	+
100			436-693	258	+
101			436-698	263	+
102			438-693	256	+
103			438-698	261	-
104	KIAA0641	76-352	1-329	329	-
105			1-334	334	-
106			1-339	339	-
107	KIAA1044	431-510	425-510	86	+
108			425-556	132	+
109			425-564	140	+
110			425-572	148	+
111			425-646	222	-
112			431-556	126	+
113			431-564	134	+
114			431-572	142	+
115			431-646	216	+
116			433-646	214	+
117			440-510	71	+
118			440-556	117	+
119			440-564	125	+
120			440-572	133	+
121			440-646	207	+
122	KIAA1131	1009-1344	998-1330	333	-
123			998-1342	345	+
124			998-1344	347	+
125			998-1353	356	+
126			1003-1342	340	+
127			1003-1344	342	+
128			1003-1353	351	-
129			1008-1330	323	+
130			1008-1342	335	+
131			1008-1344	337	+
132			1008-1353	346	+
133			1009-1330	322	-
134			1009-1342	334	-
135			1009-1344	336	+
136			1009-1353	345	+
137		1049-1344	1028-1330	303	-
138			1028-1342	315	-
139			1028-1344	317	-
140			1028-1353	326	-
141			1044-1330	287	-
142			1044-1342	299	-
143			1044-1344	301	-
144			1044-1353	310	-
145			1049-1342	294	-
146			1049-1344	296	-
147			1049-1353	305	-
148			1059-1330	272	-
149			1059-1342	284	-
150			1059-1344	286	+
151			1059-1353	295	-
152	KIAA1142	35-97	1-121	121	+
153			1-126	126	+
154			35-97	63	+
155			35-101	67	+
156			35-115	81	+
157	KIAA1256	13-117	1-117	117	+
158			13-98	86	+
159			13-108	96	+
160			13-117	105	+
161	KIAA1268	148-316	90-316	227	-
162			90-318	229	-
163			90-332	243	+
164			90-345	256	+
165			95-316	222	-
166			95-318	224	-
167			95-332	238	+
168			95-345	251	+
169			99-316	218	-
170			99-318	220	-
171			99-332	234	+
172			99-345	247	+
173			148-316	169	-
174			148-318	171	-
175			148-332	185	-
176			148-345	198	-
177	KIAA1338	180-406	165-376	212	-
178			165-395	231	-
179			165-406	242	-
180			165-414	250	-
181			169-376	208	-
182			169-395	227	-
183			169-406	238	-
184			169-414	246	-
185			172-376	205	-
186			172-395	224	-
187			172-406	235	-
188			180-376	197	-
189			180-395	216	-
190			180-406	227	-
191			180-414	235	-
192		639-861	593-861	269	+
193			593-881	289	-
194			593-889	297	-
195			593-896	304	-
196			609-861	253	+
197			609-881	273	-
198			609-889	281	-
199			609-896	288	+
200			624-861	238	+
201			624-881	258	+
202			624-889	266	-
203			624-896	273	+
204			639-861	223	+
205			639-881	243	+
206			639-889	251	+
207			639-896	258	+
208	KIAA1416	55-202	55-201	147	+
209			55-202	148	+
210			55-203	149	+
211			55-205	151	+
212	KIAA1459	745-848	745-853	109	+
213			757-848	92	+
214			765-848	84	+
215			765-853	89	+

Figure S1. List of expressed domains. Computationally dissected protein domains and their experimentally assessed solubilities. Results for the 215 fragments (encoding 24 domains) that were analyzable are shown. The Kazusa sequence identification code (KIAA code), the domain region (DOMAIN), the fragment region (FRAGMENT), the domain’s length (LENGTH), the experimentally assessed solubility (SOLUBLE) are shown. Soluble and a insoluble fragments are indicated by (+) and (-), respectively.
